# Supplementary material for: Roles of SNORD115 and SNORD116 ncRNA clusters during neuronal differentiation
Source: Nat Commun. 2024 Nov 30;15:10427. doi: 10.1038/s41467-024-54573-8 (PMC11608373; doi:10.1038/s41467-024-54573-8)
Supplement: Supplementary file 1 — Supplementary Information [file 41467_2024_54573_MOESM1_ESM.pdf]

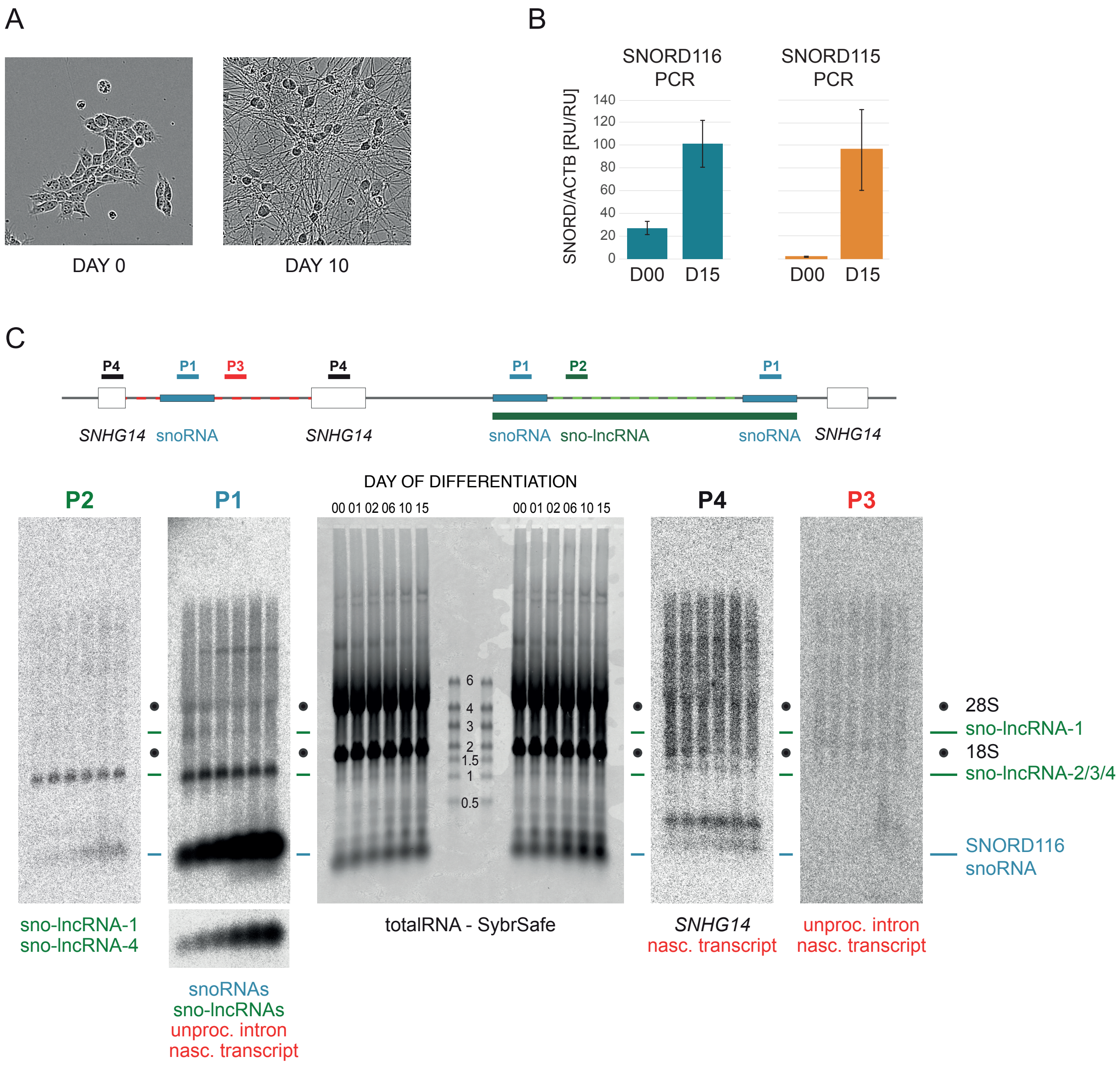

Figure S1. Differentiation of LUHMES cells.

A: Images of the differentiating wild-type LUHMES cells.

B: Relative expression of SNORD115 and SNORD116 between D00 and D15 quantified by RT-PCR analysis normalized to *ACTB* (stable upon differentiation). Error bars represent standard deviation from 3 samples from independent rounds of differentiation. Source data are provided as a Source Data file.

C: Expression of various ncRNAs originating from *SNHG14* gene by northern hybridization; P1: probe against SNORD116; P2: mix of probes against *SNHG14* introns within the sno-lncRNA; P3: mix of probes against *SNHG14* introns non-overlapping with stable ncRNAs; P4: mix of probes against *SNHG14* exons. Note that the *SNHG14* primary transcript is ~600 kb in length and cannot be detected in these analyses. This analysis was performed once. Sequences of probes listed in the Supplementary Data 9.

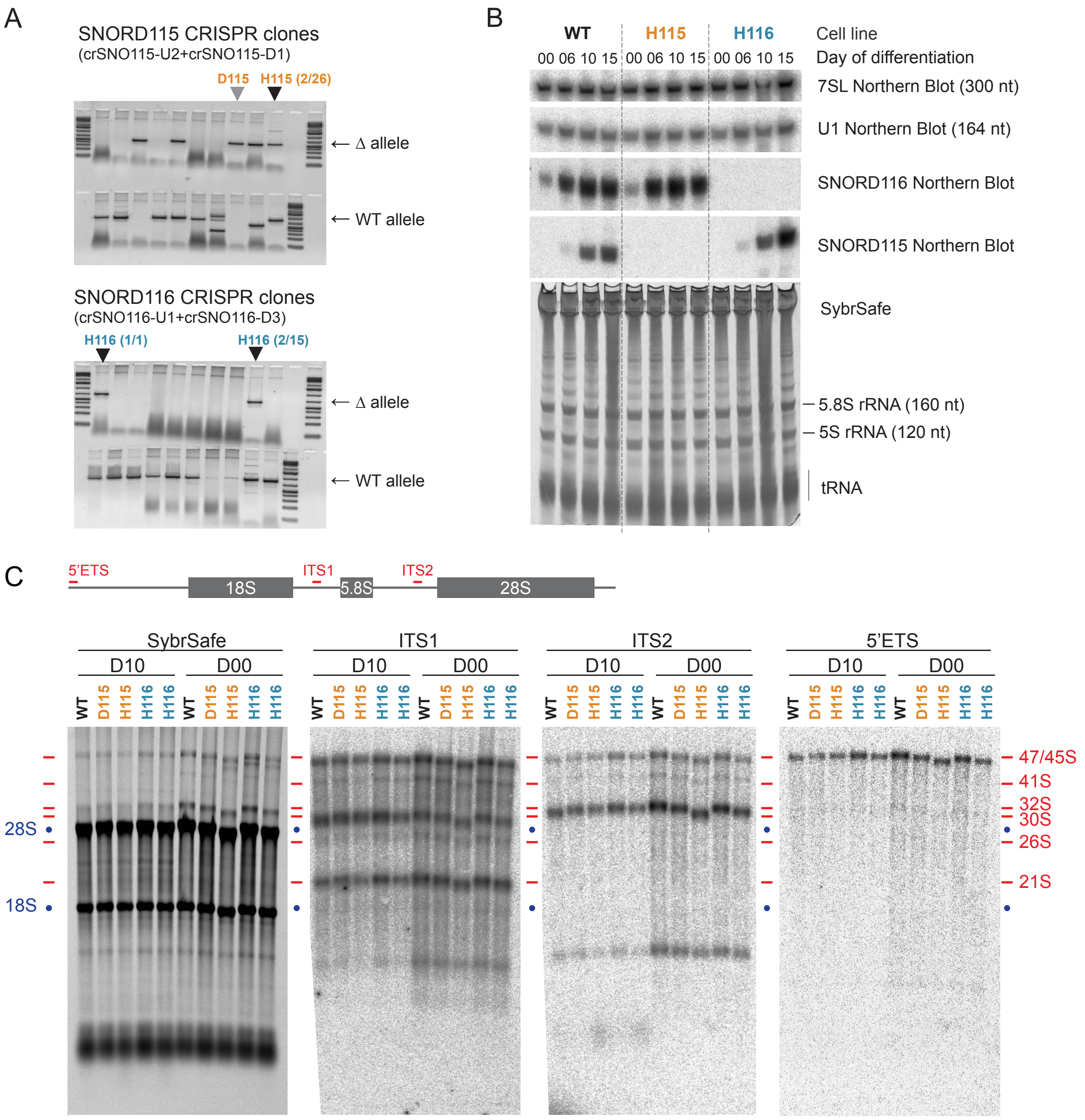

Figure S2. Construction of cell lines lacking SNORD115 of SNRORD116 expression.

A: PCR confirmation of heterozygous deletion of *SNORD115* (clone 2/12) and *SNORD116* (clones 1/1 and 2/15) clusters in the cell lines involved in the current study. Homozygous *SNORD115* deletion strain (D115) was used in the analysis of rRNA maturation only. Source data are provided as a Source Data file.

B: Time course of SNORD115 and SNORD116 expression in wildtype and mutant cell lines. Mature 5.8S rRNA, 5S rRNAs and tRNAs are visible on the scan of SybrSafe stained gel. U1 and 7SL are used as a loading control. Gel loading was normalized by total RNA. Similar profile of SNORD115 and SNORD116 expression in deletion mutants was obtained at least 3 times. Uncropped scans provided as a Source Data file.

C: Analysis of rRNA processing and accumulation. Expression of mature 28S and 18S rRNA is visible on the scan of SybrSafe stained gel. Pre-rRNA maturation was assessed by northern hybridization using probes against pre-rRNA regions indicated. ITS1 (internal transcribed spacer 1), ITS2 and 5'ETS (external transcribed spacer): 47/45S, 41S, 32S, 30S, 26S and 21S. Samples collected from wild-type and 2 independent clones of mutant cells, undifferentiated (D00) and at D10 of differentiation. Gel loading was normalized by total RNA. This analysis was performed once.

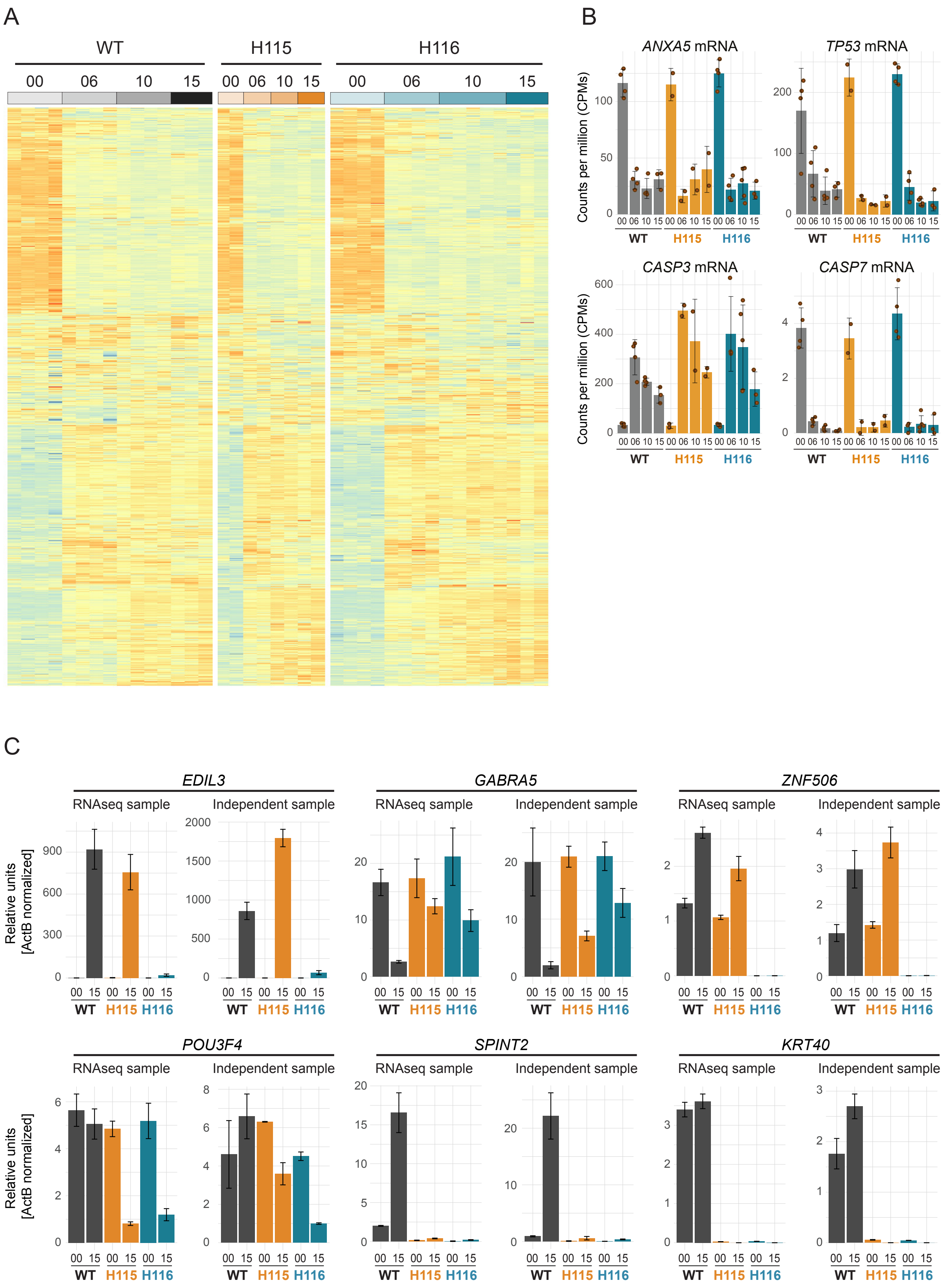

Figure S3 Altered RNA abundance during neuronal differentiation

Figure S3 Altered RNA abundance during neuronal differentiation

A: Heatmap of differentiating wild-type and mutant cells, including all RNA-seq samples with replicates.

B: Expression of cell death related genes in WT and mutant cells upon differentiation. Error bars represent standard deviation from EdgeR analysis (quasi-likelihood F-test followed by Benjamini-Hochberg correction for multitesting). Number of biological replicates for each mutant and timepoint is provided in the Supplementary Data 1.

C: RT-PCR confirmation of the RNA-seq data on a selection of genes. Each test was performed on one sample included in RNA-seq and one independent sample. Expression quantified relative to ACTB expression. Error bars represent standard deviation from technical replicates.

Source data are provided as a Source Data file.

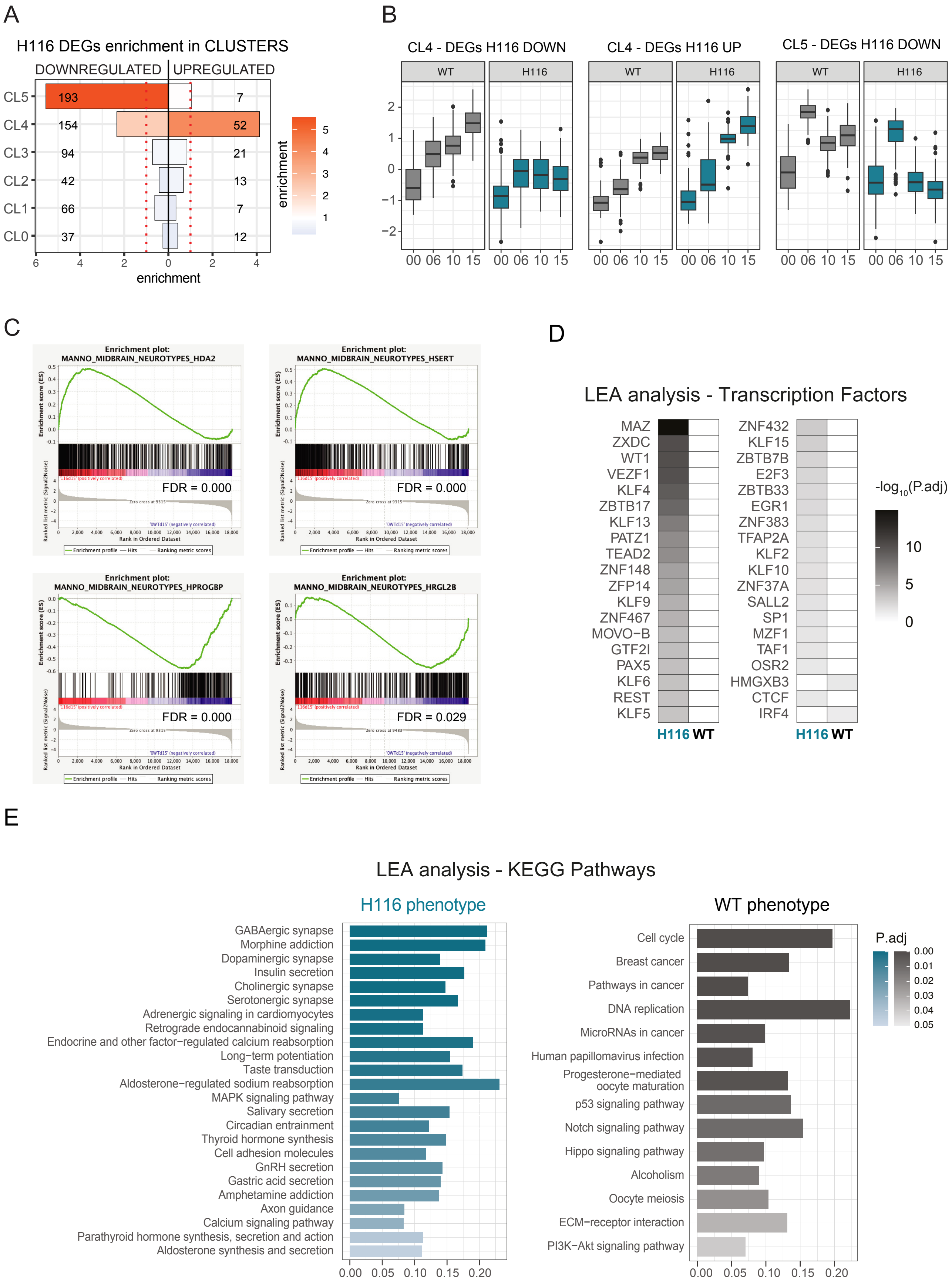

Figure S4 Pathway analyses

Figure S4 Pathway analyses
A: Enrichment of DEGs in H116 cell line, in 6 clusters defined by k-means clustering (Fig. 3), based on the expression patterns of genes in wildtype cells.
B: Pattern of expression of H116 DEGs in wildtype and mutant cells most affected by deletion, clusters 4 and 5. Distribution of mean expression levels (scaled gene-wise) for each timepoint (number of biological replicates for each cell type and timepoint is provided in the Supplementary Data 1). The center line represents the median, bounds of box - lower and upper quartile; the whiskers are reaching the largest or the smallest value, at most 1.5 IQR of the bounds, outliers are marked with the dots.
C: Example enrichment plots for cell types with similar transcriptional profile to H116 deletion mutant at day 15 of differentiation: HDA2 – dopaminergic neurons, subtype 2; HSERT – serotonergic neurons; and to wild-type LUHMES cells: HPROGBP – progenitor basal plate; HRGL2B – radial glia-like cells, subtype 2B. Plots relate to the analysis from GSEA analysis in Fig 5I. GSEA algorithm uses the in-house statistics, described in detail in the original publication (10.1073/pnas.0506580102). Midbrain single cell expression data from La Manno *et al.* (10.1016/j.cell.2016.09.027) deposited in the Molecular Signatures Database (MSigDB, 10.1073/pnas.0506580102).
D: Expression of genes responsible for “mature” phenotype of H116 cells is associated with the activity of transcription factors with high significance. There is no similar association with the genes responsible for wildtype phenotype. Genes associated with phenotypes were identified by GSEA/LEA analysis. Transcription factor enrichment analyzed by g::Profiler using default statistical parameters: Fishers’ one-tailed test and multiple testing correction algorithm g:SCS. Detailed outcome of the analysis is in the Supplementary Table 5.
E: KEGG pathways enrichment analysis of the LEA identified genes, responsible for separation between H116 and wild-type phenotypes, as analyzed by g::Profiler (statistical parameters as above). Detailed outcome of the analysis is in the Supplementary Table 5.
Source data are provided as a Source Data file.

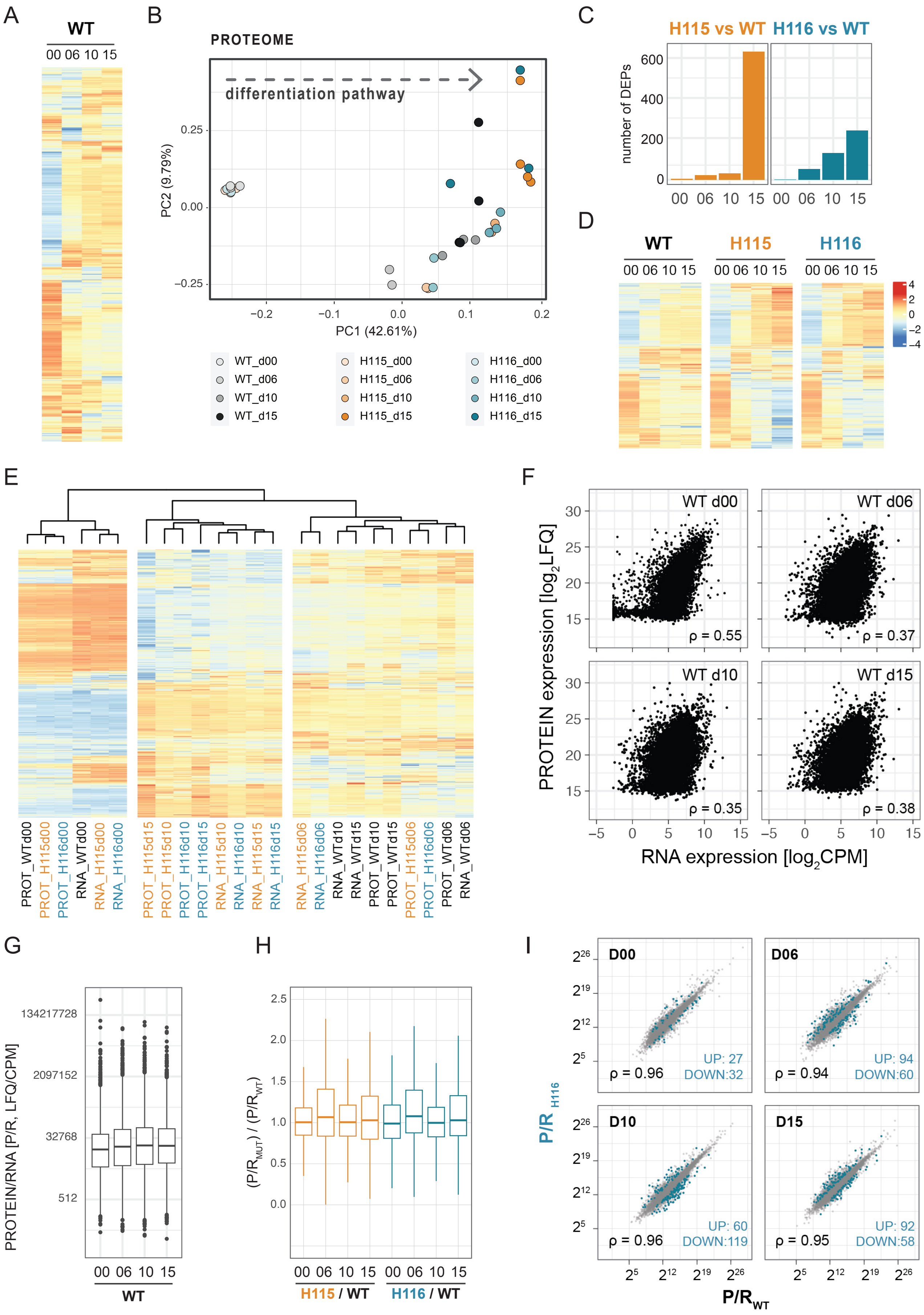

Figure S5. Transcriptome-proteome analysis.

Figure S5. Transcriptome-proteome analysis.

A: Expression of all quantified proteins across differentiation process in wild-type cells.

B: PCA analysis of proteomic data supports accelerated differentiation of H115 and H116 cells observed in the PCA analysis of transcriptomic data (Fig. 5H) and comparison with scRNA-seq on differentiating human midbrain (Fig. 5**I**).

C: Number of differentially expressed proteins in each mutant cell line relative to WT increases with the course of differentiation.

D: Pattern of expression of all identified differentially expressed proteins (DEPs) in wild-type and mutant cells.

E: Proteome and transcriptome samples cluster together. Clustering performed on independently scaled proteomic [log_2LFQ] and transcriptomic data [log_2CPM] for differentially expressed proteins.

F: Relation between transcript [CPM] and protein [LFQ] levels in differentiating LUHMES cells. Spearman correlation (*ρ*) is higher in cycling than differentiating cells.

G: The steady-state amount of protein per steady-state amount of transcript (P/R ratio) is highly variable. Distribution of P/R ratios for each timepoint calculated for WT cells, exact number of replicates is provided in the Supplementary Data 1. The center line represents the median, bounds of box - lower and upper quartile; the whiskers are reaching the largest or the smallest value, at most 1.5 IQR of the bounds, outliers are marked with the dots.

H: P/R ratio is a characteristic feature of a gene at a given differentiation stage and is generally maintained between wild-type and mutant cells. Box parameters same as for panel G. This plot does not contain all te values and is zoomed in to show the narrow interquartile range around 1. Outliers are removed for clarity.

I: Correlation of P/R ratios between WT and H116 during differentiation. Higher and lower values were observed in the mutant. *ρ*, Spearman correlation. Source data are provided as a Source Data file.

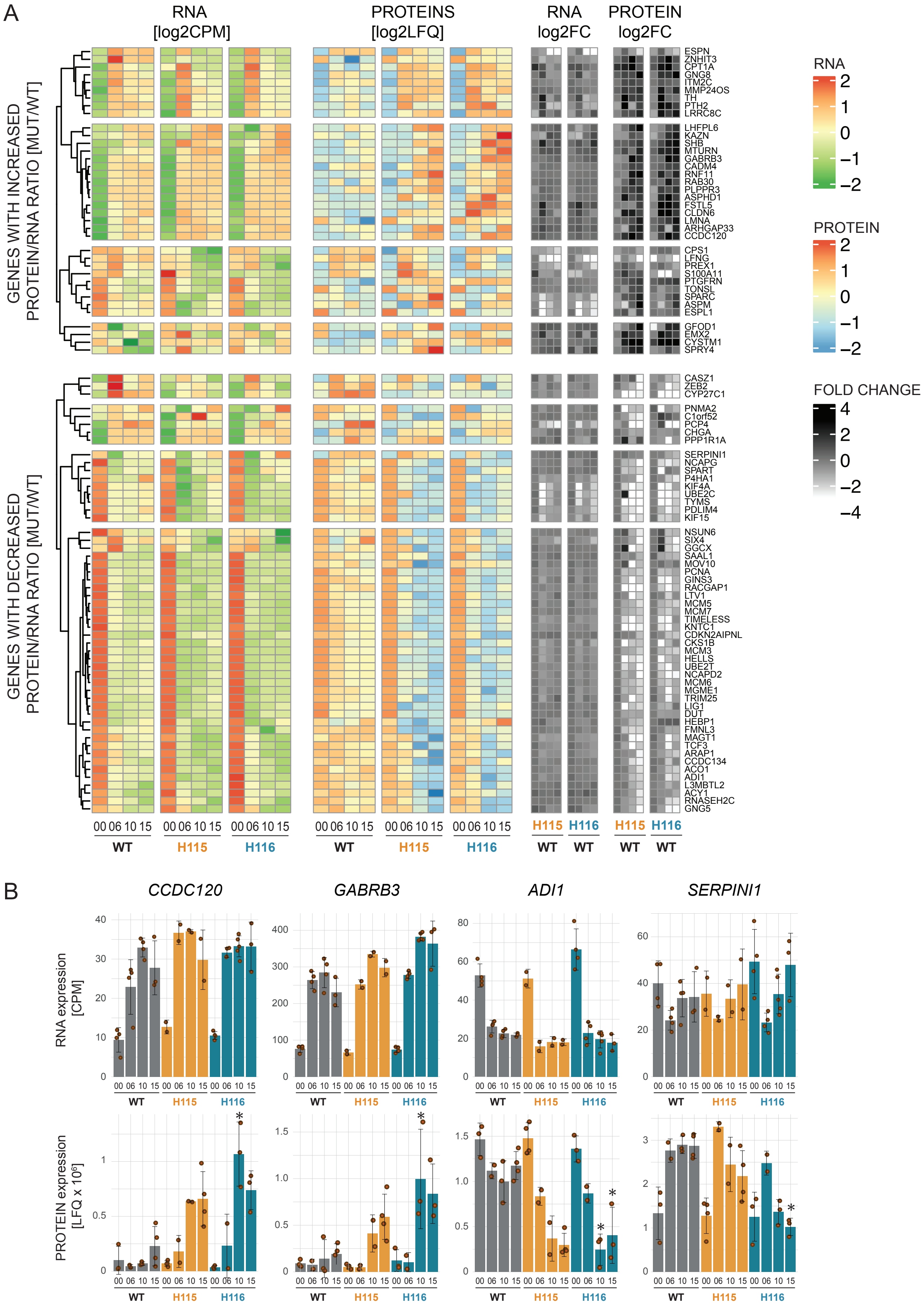

Figure S6. Post-transcriptional effects of *SNORD115* and *SNORD116* clusters loss.

Figure S6. Post-transcriptional effects of *SNORD115* and *SNORD116* clusters loss.
A: Combined analysis of transcriptome and proteome data revealed different relation between protein and mRNA expression in wildtype vs mutant cells. Protein/RNA (P/R) ratio can be either decreased or increased, and potentially indicating the involvement of *SNORD115* and *SNORD116* clusters in post-transcriptional regulation of gene expression.
B: mRNA and protein expression plots for selected genes displaying altered P/R ratio in wildtype and mutant cells. Marked statistical significance for the difference in expression between mutant and WT cell lines from EdgeR (FDR; quasi-likelihood F-test followed by Benjamini-Hochberg correction for multitesting) and DEP analyses (empirical Bayes statistics followed by Benjamini-Hochberg correction for multitesting); error bars represent standard deviation. Source data are provided as a Source Data file.

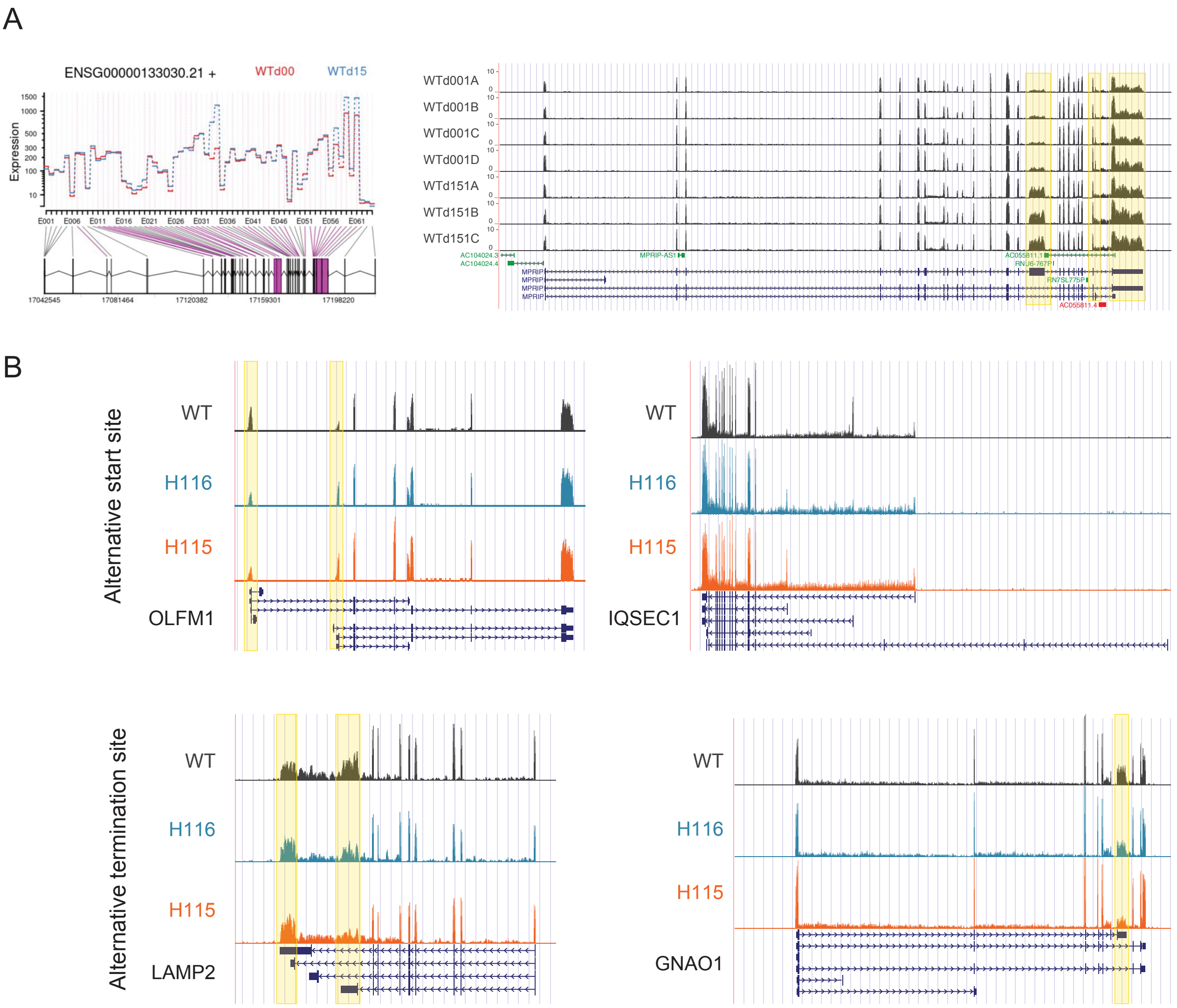

Figure S7. Deletion strains H115 and H116 show minimal splicing defects, but altered transcription start and end sites.

A: *MPRIP* gene changes splice variants upon differentiation.

B: Examples of genes with alternative exon usage identified by DEX-seq (chi-square statistical test, followed by Banjamini-Hochberg correction for multitesting) display alternative start and termination sites, instead of expected alternative splice variants.

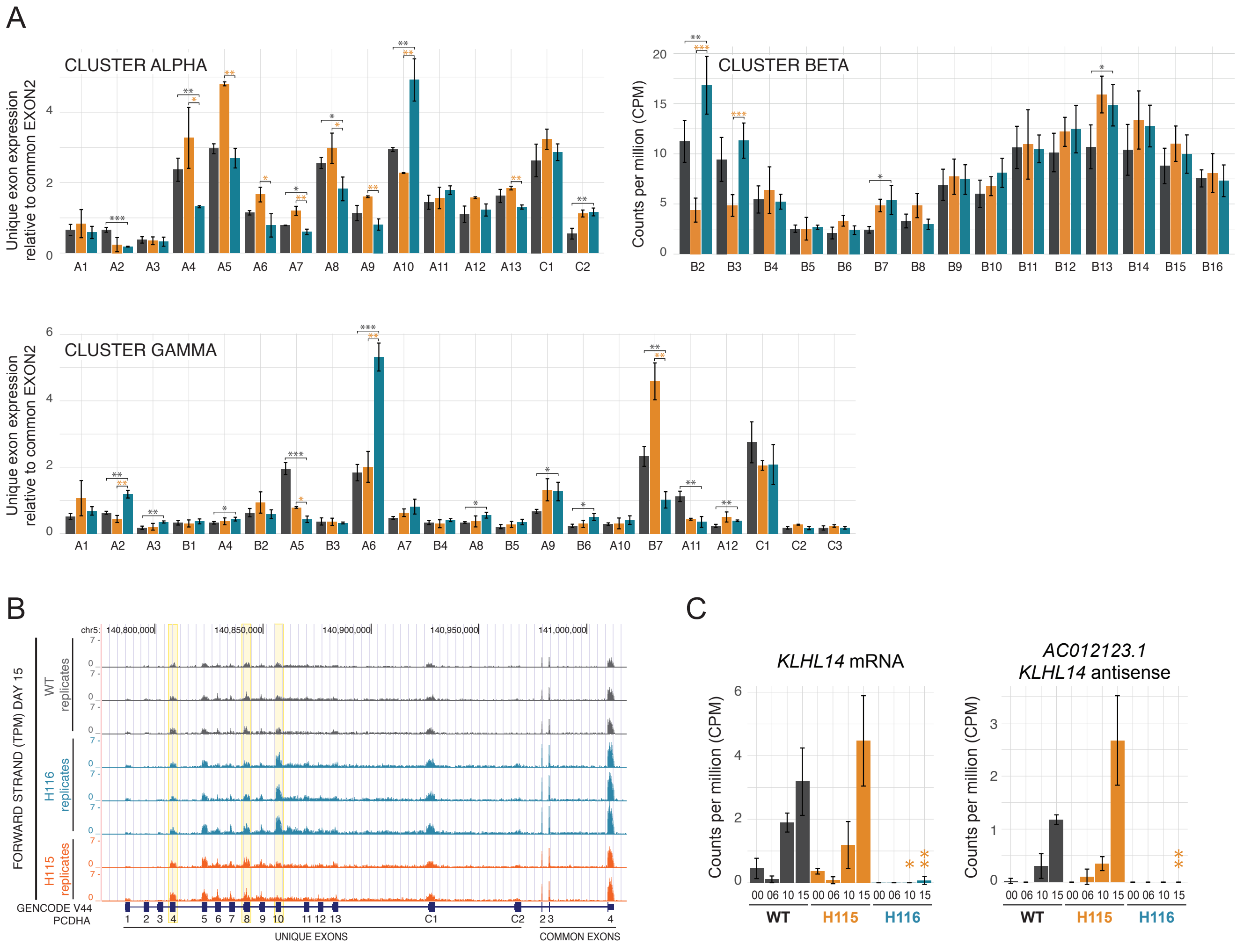

Figure S8. Transcriptional changes to SNORD116-specific genes.

A: Relative expression of clustered Protocadherin genes alpha, beta and gamma in wild type and mutant cells at D15 of differentiation. For genes from alpha and gamma clusters expression of the unique exon 1 is normalized to the expression of common exon 2 to account for the difference in expression of the whole cluster. For cluster beta formed by mono-exonic genes, counts per million (CPM) from the EdgeR are used. Error bars represent standard deviation (SD), statistical significance (p-value) for clusters alpha and gamma tested with Student t-test, for cluster beta p-value originates from EdgeR analysis (quasi-likelihood F-test). Number of biological replicates for each cell line and timepoint is provided in the Supplementary Data 1.

B: UCSC genome browser view of transcription across clustered Protocadherin genes alpha in wild-type and mutant cell lines at D15.

C: Mean expression of *KLHL14* mRNA and antisense non-coding transcript across differentiation. Marked statistical significance for the difference in expression between H116 and H115 cell lines originates from EdgeR analysis (quasi-likelihood F-test followed by Benjamini-Hochberg correction for multitesting). Error bars represent standard deviation. Source data are provided as a Source Data file.

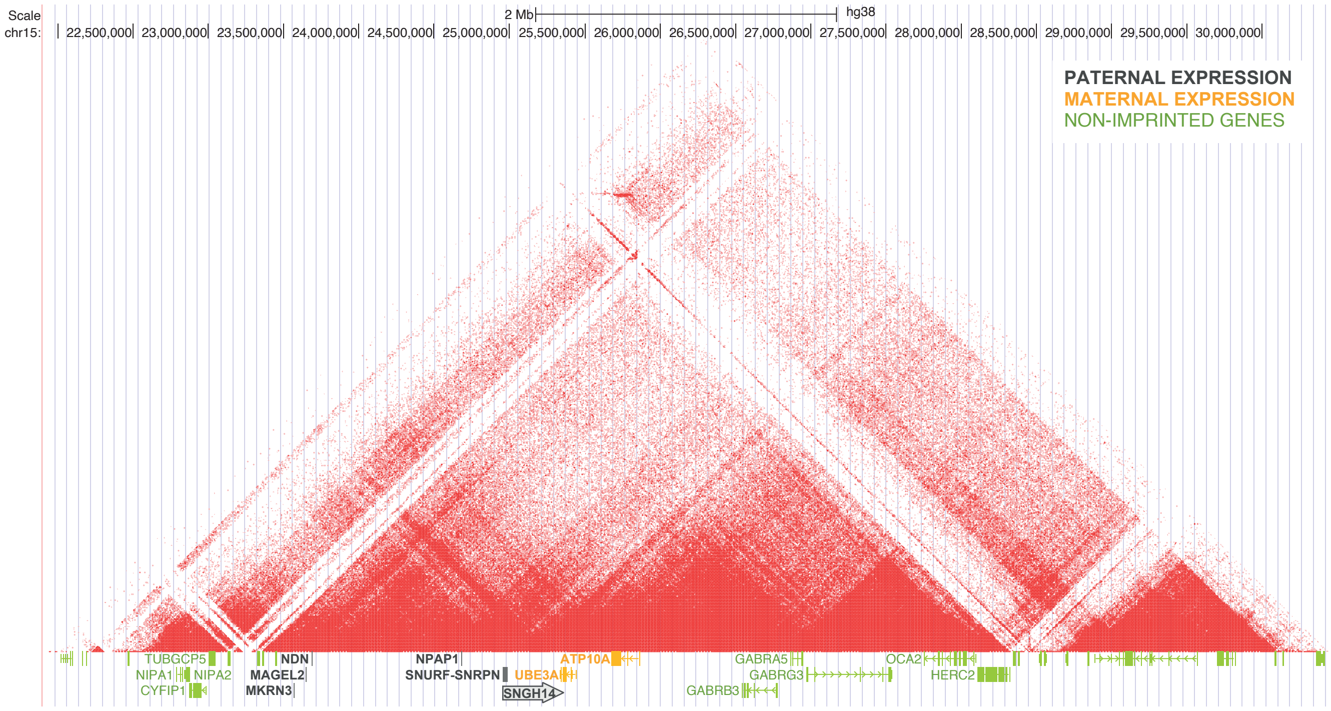

Figure S9. Chromatin structure of the *PWS* locus.

UCSC genome browser view of the chromatin structure of the *PWS* locus obtained from Micro-C XL experiments on the embryonic stem cells H1-hECS. Color scheme as in Figure 1; grey, paternally expressed genes; yellow, maternally expressed genes; green, non-imprinted genes.
