# Supplementary material for: Roles of SNORD115 and SNORD116 ncRNA clusters during neuronal differentiation
Source: Nat Commun. 2024 Nov 30;15:10427. doi: 10.1038/s41467-024-54573-8 (PMC11608373; doi:10.1038/s41467-024-54573-8)
Supplement: Supplementary file 2 — Description of Additional Supplementary Files [file 41467_2024_54573_MOESM2_ESM.pdf]

Description of Additional Supplementary Files

Supplementary Data 1: List of the RNAseq and Mass Spectrometry samples, Spearman correlation between RNAseq samples and similarity of EdgeR and limma analyses.

Supplementary Data 2: Detailed outcome of the GO term analysis of clusters.

Supplementary Data 3: Differentially expressed genes from RNAseq data analysis.

Supplementary Data 4: Detailed outcome of the GO term analysis of DEGs between H116 and wildtype cells.

Supplementary Data 5: Transcription factors and KEGG pathway analysis of genes associated with “mature” phenotype of H116 cells.

Supplementary Data 6: Differentially expressed proteins from Mass Spectrometry data analysis.

Supplementary Data 7: Expression of SNORD116-specific genes from RNA-seq data analysis and potential SNORD116 binding sites on the SNORD116-specific genes and processed mRNAs as predicted by PLEXY.

Supplementary Data 8: Non-exhaustive list of the publications supporting links between SNORD116-specific genes and PWS phenotype.

Supplementary Data 9: List of oligonucleotides for CRISPR, PCR and Northern blot.
